# MicroRNA Transcriptome of Poly I:C-Stimulated Peripheral Blood Mononuclear Cells Reveals Evidence for MicroRNAs in Regulating Host Response to RNA Viruses in Pigs

**DOI:** 10.3390/ijms17101601

**Published:** 2016-09-22

**Authors:** Jiying Wang, Yanping Wang, Haifei Wang, Jianfeng Guo, Huaizhong Wang, Ying Wu, Jianfeng Liu

**Affiliations:** 1 Shandong Provincial Key Laboratory of Animal Disease Control and Breeding, Institute of Animal Science and Veterinary Medicine, Shandong Academy of Agricultural Sciences, Jinan 250100, China; jnwangjiying@163.com (J.W.); wangyanping03@163.com (Ya.W.); guojf982@163.com (J.G.); whzh0825@163.com (H.W.); wusaas@163.com (Yi.W.); 2 Key Laboratory of Animal Genetics, Breeding and Reproduction, Ministry of Agriculture, College of Animal Science and Technology, China Agricultural University, Beijing 100193, China; wanghaiffei@126.com

**Keywords:** poly I:C, PBMC, miRNA-seq, target genes, pigs

## Abstract

MicroRNAs (miRNAs) are one family of small noncoding RNAs that function to modulate the activity of specific mRNA targets in animals. To understand the role of miRNAs in regulating genes involved in the host immune response to RNA viruses, we profiled and characterized the miRNAs of swine peripheral blood mononuclear cells (PBMC) stimulated with poly I:C, a synthetic dsRNA analog, by miRNA-sequencing (miRNA-seq). We identified a total of 905 miRNAs, of which 503 miRNAs were firstly exploited herein with no annotation in the latest miRBase 21.0. Expression analysis demonstrated that poly I:C stimulation can elicit significantly differentially expressed (DE) miRNAs in Dapulian (*n* = 20), one Chinese indigenous breed, as well as Landrace (*n* = 23). By integrating the mRNA expression profiles of the same sample with miRNA profiles, we carried out function analyses of the target genes of these DE miRNAs, with the results indicating that target genes were most enriched in some immune-related pathways and gene ontology (GO) terms, suggesting that DE miRNAs play an important role in the regulation of host to poly I:C stimulation. Furthermore, we also detected 43 and 61 significantly DE miRNAs between the two breeds in the control sample groups and poly I:C stimulation groups, respectively, which may be involved in regulation of the different characteristics of the two breeds. This study describes for the first time the PBMC miRNA transcriptomic response to poly I:C stimulation in pigs, which not only contributes to a broad view of the pig miRNAome but improves our understanding of miRNA function in regulating host immune response to RNA viruses.

## 1. Introduction

The domestic pig (*Sus scrofa*) is one of the most important meat-producing livestock species worldwide. Besides their importance in livestock production, pigs are excellent animal models to investigate various infectious diseases because of their phylogenetic relation to humans [1]. In pig production, viral or bacterial pathogens can cause various infectious diseases, significantly decreasing production efficiency and bringing huge economic losses. Selection of animals for improved disease resistance or resilience is of major economic importance. Previous studies have demonstrated that infectious disease resistance capability substantially varied across individuals as well as populations [2,3]. Moreover, indigenous pig breeds are generally more resistant than modern commercial pig breeds [4,5]. However, the genetic basis and immune mechanism of their difference remain largely unknown so far. Thus, unravelling the genes, regulators, and networks that control porcine immune responses and contribute to disease-resistance phenotypes has become more and more important.

MicroRNAs (miRNAs) are found as one family of short (typically 22 nt in size), endogenous, non-coding RNAs that function to modulate gene regulation via pairing to the specific mRNA targets in genomes of animals and plants, further affecting their posttranscriptional repression [6]. Cumulative evidence demonstrated that miRNAs are involved in a broad range of biological processes, ranging from development to metabolic regulation and immunization [7]. Furthermore, it has been suggested that miRNAs could be promising biomarkers for pathologic diagnosis and prognosis due to their stability in biofluids [8,9,10]. Recently, many studies have been directed to pig tissue-specific miRNA repertoires using high-throughput sequencing. However, compared with human and other model animals, limited miRNAs have been identified in pigs, especially those generated in the process of immune regulation.

Peripheral blood mononuclear cells (PBMC), isolated from whole blood cells, are clinically relevant cells that display a variety of both innate and adaptive immune functions, and have been extensively used as an in vitro model to dissect the pathogenesis and genetics behind infection or stimulation [11,12,13,14]. Polyinosinic: polycytidylic acid (poly I:C) is a synthetic viral double-stranded RNA (dsRNA) analog. Previous studies in human and other species have demonstrated that it can mimic viral infection, and has stimulatory effects similar to viral dsRNA [15,16,17]. Using poly I:C as an immunologic stimulant, gene transcriptome analyses of PBMC or whole blood have made tremendous progress in humans as well as in pigs [18,19,20]. However, research on the miRNA regulation generated in porcine PBMC with poly I:C stimulation is currently lacking. To better understand the regulatory role of miRNAs in the porcine model, it is critical to identify miRNAs expressed in PBMC and discover the differentially expressed (DE) miRNAs of PBMC in response to poly I:C stimulation.

With the application of high-throughput sequencing technology, miRNA-sequencing (miRNA-seq) takes advantage of both detecting low-expression miRNAs and predicting unannotated miRNAs, enabling comprehensive analysis of the host miRNA transcriptome to pathogen infection or immunostimulant. To elucidate the mechanism of miRNAs regulation in the host transcriptional response to poly I:C stimulation, we stimulated the PBMC of piglets coming from one modern commercial breed (Landrace) and one Chinese indigenous breed (Dapulian) with poly I:C by miRNA-seq, detected the miRNAs which involved in the response of the host to poly I:C stimulation. Moreover, we also compared the miRNA expression profiles of PBMC between the two breeds. This study describes for the first time the PBMC miRNA transcriptomic response to poly I:C stimulation in pigs, identifies many miRNAs specifically expressed in PBMC, and provides crucial evidence for exploring the role of miRNAs in the response to RNA viruses, and the potential molecular mechanisms of different genetic resistance to viral infection among different pig breeds.

## 2. Results

### 2.1. An Overview of the Sequencing Results

Using Illumina single-read sequencing, we sequenced the miRNA transcriptome of PBMC, stimulated with poly I:C for 24 h or only in vitro cultured for 24 h. In total, we generated 9.93–17.82 million raw reads per sample. The raw reads were then subjected to a series of data filtration steps to remove adapter dimers, low complexity, common RNA families, and repeats, and clean reads meeting the acceptance criteria were termed mappable sequences and used in further miRNA identification analysis. The statistics for the distribution of small RNAs during a series of filters is shown in Table S1. On average, 8.81 million (68.37% of the total reads) mappable sequences per sample were produced, which represented most of the total number of sequences.

**Figure 1:**
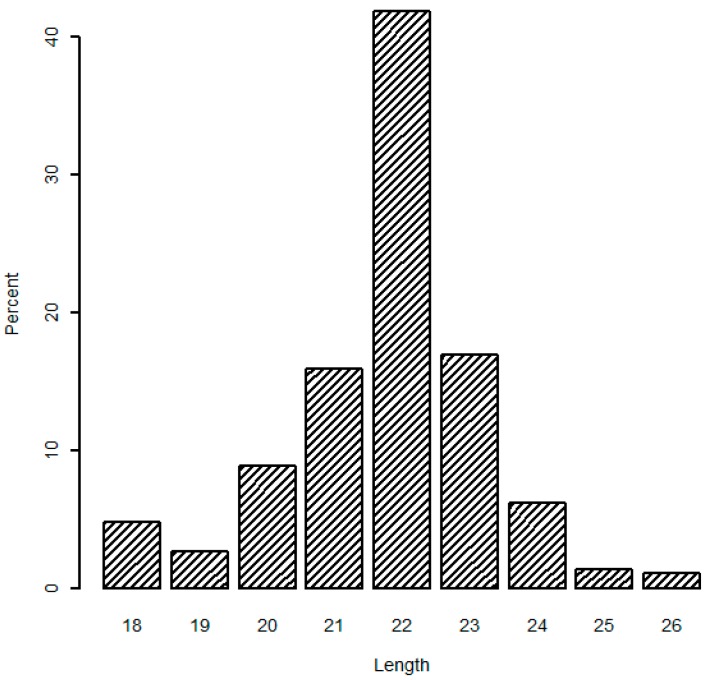
The length distribution of mappable sequences.

In addition, the analysis of mappable sequences illustrated that the length distribution peaked at 22 nt (average 41.82%), and 21 and 23 nt sequences accounted for 74.75% of the total mappable sequences (Figure 1), which are typical products of Dicer incisions. Similar variation in miRNA length has been repeatedly detected in deep sequencing results of other studies [21,22]. Both the read number and length distribution of mappable sequences confirmed that our constructed RNA libraries were of high quality and the sequence data reached the criteria for follow-up analyses.

### 2.2. Known and Novel miRNA Identification

**Figure 2:**
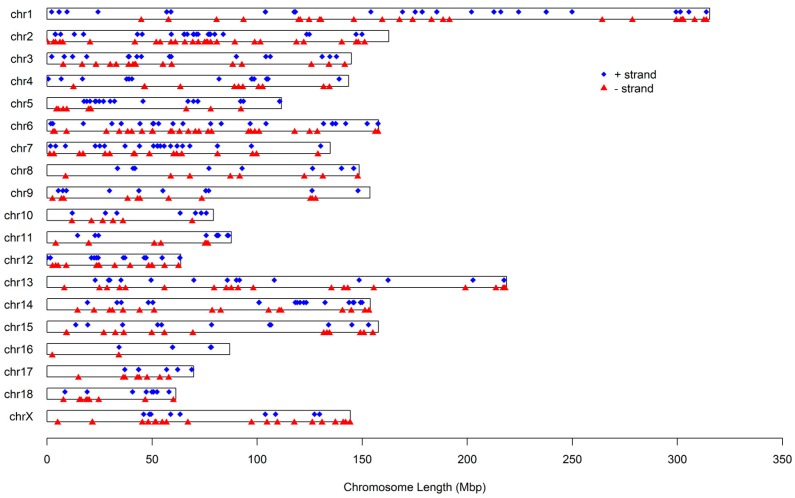
Distribution of miRNAs identified in the study on the chromosomes. Blue diamonds and red rectangles mean + strand and − strand, respectively.

MicroRNA identification was performed by a proprietary pipeline script, ACGT101-miR v4.2 (LC Sciences, Houston, TX, USA), as specified in the Material and Methods section. In total, we identified 784 pre-miRNAs, which expressed 905 mature miRNAs. Detailed information on the expressed miRNAs and the summary of their length distribution are presented in Tables S2 and S3, respectively. We found that the lengths of these miRNAs were distributed in a similar manner to the mappable sequences, with the majority being between 21 and 23 nt. Most of the miRNAs identified had an exact genome location, whereas there were 192 miRNAs (21.22%) whose chromosomal locations were not determined because of the uncomplete genome sequence of Sscrofa10.2. Figure 2 illustrates the distribution of these miRNAs on the 18 autosomes and X chromosome. It can be seen that these miRNAs are not uniformly distributed among chromosomes. The number proportion of miRNAs on chromosomes varied from 0.94% to 10.69%, with chromosome Xharboring the greatest number of miRNAs and chromosome 16 having the fewest. Additionally, the number of miRNA loci on a chromosome′s positive and negative strands was approximately equal, at 341 and 373, respectively.

Compared with miRBase 21.0, these miRNAs were further divided into three types, “yes”, “diff”, and “new”. One hundred seventeen miRNAs belonged to “yes”, confirming miRNA sequences in miRBase; 285 belonged to “diff”, confirming miRNA sequences in miRBase, but different sequences (isoform) were reported in our study; and 503 belonged to “new”, miRNAs identified in this study and not reported in miRBase 21.0. Our results indicated that most of the miRNAs identified herein are reported for the first time, and some may be specifically expressed in PBMC.

According to the comparison with average expression level of all miRNAs identified, the 905 unique miRNAs were further divided into high (higher than the average), middle (higher than 10 copies and less than average), and low groups (less than 10 copies), which contained 51, 358, and 496, respectively. High count miRNAs accounted for only 5.64% of the total unique miRNAs identified, but for 96.40% of the total miRNA expression, suggesting that a few miRNAs comprised the majority of sequences and these miRNAs may be involved in the basic functions of PBMC. Similar distribution of high count miRNAs has been repeatedly observed in deep sequencing results of other studies [21,22,23].

On closer examination of the 905 miRNAs identified, it can be found that the great majority of them (753 miRNAs, 83.20%) were co-expressed in both the poly I:C stimulation groups and control groups, whereas there were 152 miRNAs (16.80%) only expressed in the stimulation groups or control groups. On the other hand, there were 733 miRNAs (81.00%) co-expressed in both Dapulian and Landrace breeds, whereas 172 miRNAs (19.00%) were only expressed in one of the two breeds. Furthermore, comparing the miRNA expression of the control groups and stimulated groups, there was a little higher miRNA expression in the control groups than the poly I:C stimulation groups (4418.94 vs. 4298.43). On the other hand, higher miRNA expression was observed in Landrace than Dapulian (4665 vs. 3987). However, the expression differences between the two group as well as the two breeds did not reach statistical significance by paired sample *t*-test.

**Figure 3:**
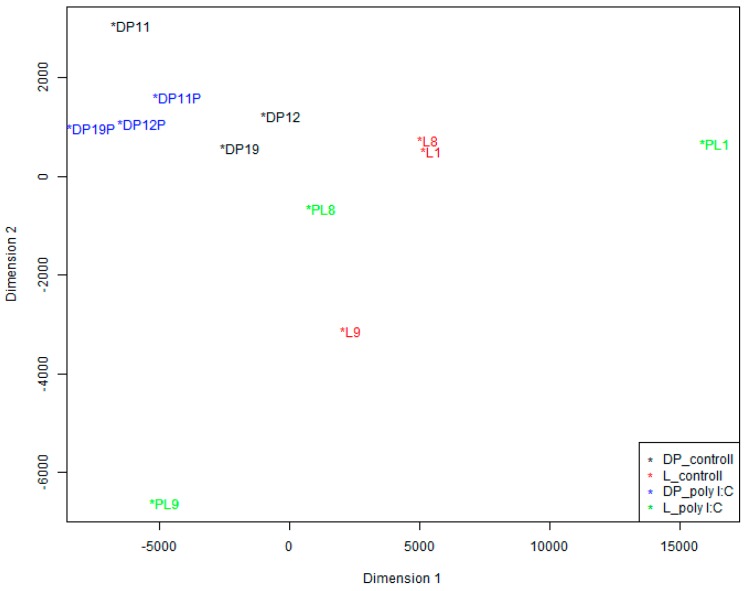
Multidimensional Scaling (MDS) analysis of the control and poly I:C stimulated samples using expression of all miRNAs. Black, red, blue and green dots mean samples of DP_controll, L_controll, DP_poly I:C, and L_poly I:C, respectively.

We also conducted a Multidimensional Scaling (MDS) analysis to visualize how the data cluster. As shown in Figure 3, poly I:C challenge can stimulate PBMCs, and there was much change in the coordinate position between the control and poly I:C samples of the same pig. Furthermore, we can see that there were large coordinate difference between the two breeds in the control sample groups as well as in the poly I:C stimulation sample groups. However, there is substantial inter-individual variation between pigs within breeds.

### 2.3. Differentially Expressed miRNAs in Response to Poly I:C Stimulation and Their Function Analyses

Differential expression analysis between the poly I:C stimulation group and control group were conducted for the two breeds separately. The complete lists of DE miRNAs (*p*-value < 0.05) are given in Table S4. In Dapulian, there were 20 significantly DE miRNAs detected, of which 12 miRNAs were upregulated and eight were downregulated after poly I:C stimulation. In Landrace, there were 23 significantly DE miRNAs observed, with 13 upregulated and 10 downregulated ones. However, further comparing the two DE miRNA lists, no significantly DE miRNAs were shared by the two breeds.

To gain insight into the function of the DE miRNAs detected, we predicted the potential target genes of these miRNAs using TargetScan and miRanda. We also sequenced the mRNA expression profiling of the same samples selected for miRNA profiling in the study using mRNA-seq [20]. Thus, we integrated the mRNA and the miRNA expression profiles by matching the datasets obtained for each samples to refine the in silico target genes. Consequently, only those target genes inversely expressed to DE miRNAs were selected for further functional analyses. Detailed information about the target genes is provided in Table S5.

Pathway and gene ontology (GO) enrichment analyses demonstrated that there were 26 and 18 significant pathways with *p*-value <0.05 detected after Bonferroni correction in Dapulian and Landrace, respectively. Furthermore, comparing the two pathway lists, we found 10 common significantly overrepresented pathways; detailed information on pathways is given in Table 1. A lot of pathways were involved in immune response, including T cell activation (P00053), B cell activation (P00010), Toll receptor signaling pathway (P00054), Interleukin signaling pathway (P00036), inflammation mediated by chemokine, and cytokine signaling pathway (P00031). On the other hand, in the GO enrichment analysis, 293 and 182 GO terms with adjustment *p*-value <0.05 were detected after Bonferroni correction (Tables S6 and S7). Most of these terms were involved in the regulation of the metabolic process, cellular process, and apoptotic process. However, part of these significant enriched pathways were involved in immune-related process, such as “regulation of response to stimulus (GO:0048583)”, “regulation of response to stress (GO:0080134)”, and “regulation of immune system process”.

**Figure 4:**
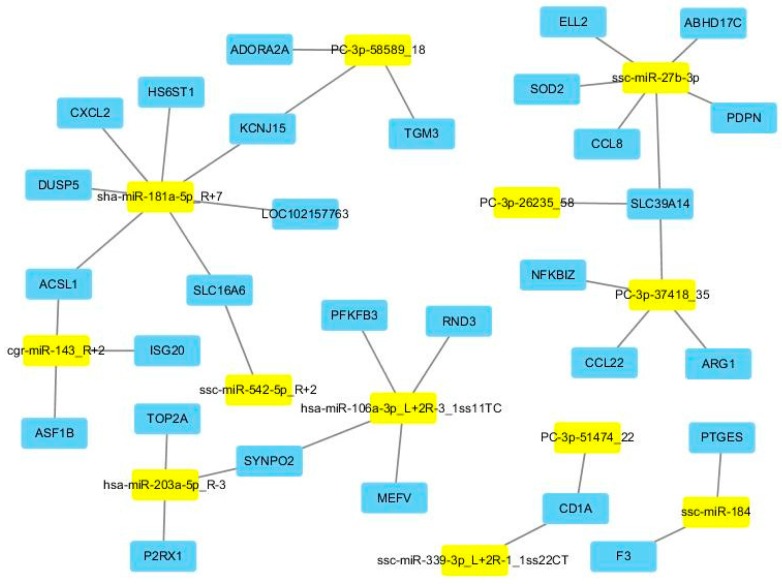
Regulation network between DE miRNAs and their DE target genes in response to poly I:C stimulation. Yellow and blue colors mean miRNAs and target genes, respectively.

For some of the miRNAs, only one target was predicted, but most miRNAs targeted multiple genes. Out of the multiple genes, some were also differentially expressed in our mRNA transcriptome analysis. Consequently, we also selected the differentially expressed target genes (DE target genes) for every DE miRNA. As shown in Table S8 of the Supplementary Materials, there were 48 miRNA-mRNA pairs identified. Figure 4 demonstrates the regulation network between DE miRNAs and their DE target genes. Additionally, for these DE target genes, four enriched GO terms were revealed after Bonferroni adjustment. More importantly, all four enriched GO terms were immune related, including cellular response to cytokine stimulus (GO:0071345), response to cytokine (GO:0034097), defense response (GO:0006952), and inflammatory response (GO:0006954).

### 2.4. Differentially Expressed miRNAs between Breeds, and Their Function Analyses

Using the same method and criterion, we also compared the miRNA expression profile of PBMC between the two breeds, including the control sample groups (DC vs. LC) and the poly I:C stimulation sample groups (DT vs. LT). The complete lists of DE genes are listed in Table S9. For the control groups (DC vs. LC) of the two breeds, i.e., PBMC cultured 24 h only, 43 DE miRNAs were detected, of which 15 miRNAs were upregulated and 28 were downregulated. On the other hand, for the poly I:C stimulated group, i.e., PBMC with poly I:C stimulated 24 h, 61 DE miRNAs were observed, with 39 upregulated and 22 downregulated ones. Comparing the DE miRNAs of the two groups, there were nine overlaps between the two lists, including three upregulated and six downregulated ones.

Similarly, the target genes of these DE miRNAs (Table S10) were firstly predicted using TargetScan and miRanda, and then refined by the mRNA expression of these target genes. Pathway and GO enrichment results are presented in Tables S11–S13. A total of 28 pathways with adjusted *p*-value <0.05 were observed in the control groups and poly I:C stimulation groups, and 11 of these pathways were consistent in both the control and the stimulation groups. Additionally, in the GO enrichment analysis, 245 and 444 GO terms with adjusted *p*-value <0.05 were identified.

### 2.5. Quantitative Real-Time PCR Validation

**Figure 5:**
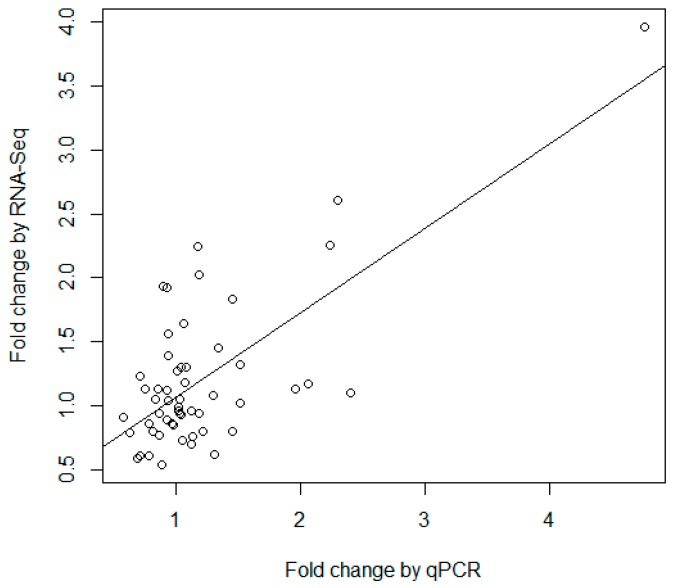
Correlation analysis of the fold changes between qPCR and RNA-seq.

More than half of the miRNAs identified in the study were reported for the first time. In order to assess the reliability of the identified miRNAs, nine miRNAs representing different expression levels were chosen to be validated by quantitative real-time PCR (qPCR) using the miScript PCR System (Qiagen, Hilden, Germany). Ssc-miR-34a and Ssc-miR-107 were selected as endogenous controls, which were confirmed to be stably expressed in the PBMC [24]. For each of the nine miRNAs tested and two endogenous controls, one miRNA-specific primer was designed, as detailed in Table S14. Standard curves were generated by using a pooled cDNA mixture, and the PCR amplification efficiencies of all primers were determined with results between 0.9 and 1.1. All δ Ct and statistical results of these qPCR tests are available in Table S15. Some of the validated miRNAs were significantly differently expressed in DT vs. DC or LT vs. LC, while the other ones were not. So instead of comparing the significant value obtained by the two methods, in the study correlation analysis of the fold change between qPCR and RNA-seq (Table S16) was used to compare the consistency of the two techniques. As demonstrated in Figure 5, the fold changes measured by RNA-seq and qRT-PCR were significantly correlated (*p*-value = 2.45 × 10^−9^) with a correlation coefficient of 0.70. The results demonstrated that there was a general consistency between qPCR and high-throughput sequencing, though there were some differences in the results of the two different technologies. Previous studies have indicated that RNA-seq is an accurate and powerful tool to identify new miRNAs and quantify miRNA expression [25,26]. On the other hand, the small size and heterogeneity of microRNAs present challenges for detection by RT-qPCR. A range of parameters is critical to the design of a successful PCR assay, such as designing specific primers, harmonizing melting temperatures, and avoiding formation of dimers [27]. These may be some of the possible reasons for the inconsistency between qPCR and miRNA-seq.

## 3. Discussion

In resisting viral infection, the immune system is the most crucial barrier. Although great progress has been achieved in mining mammalian immune-related genes, little is known about the role of miRNAs in the host immune response to viruses. To gain insight into miRNAs′ regulation of the host transcriptional response to poly I:C, a synthetic dsRNA analog, in the present study we sequenced the PBMC of piglets with poly I:C stimulation using miRNA-seq, identified 402 known and 503 novel miRNAs, detected a batch of DE miRNAs in response to poly I:C stimulation and the difference between the two breeds, and analyzed these DE miRNAs′ regulatory roles. This study describes for the first time the PBMC miRNA transcriptomic response to poly I:C stimulation in pigs, which not only contributes to a broad view of the pig miRNAome but also improves understanding of miRNA function in regulating host immune response to RNA viruses.

Differential expression analysis showed that poly I:C challenge can stimulate 20 and 23 significantly DE miRNAs in Dapulian and Landrace, respectively. Consistently with gene expression analysis [20], our results demonstrated that PBMC samples challenged with poly I:C can elicit certain miRNA expression changes, proving the immunologic stimulant applied herein, which could potentially permit in vitro screening of pigs for optimal innate immune responsiveness. However, no significant DE miRNAs were shared between the two breeds. Apart from the small sample size used in the study, one explanation for the seemingly inconsistent results is the difference between the two breeds. Compared with modern commercial breeds, Dapulian, an indigenous pig breed raised in North China, exhibits excellent performance in meat quality and disease resistance, especially against porcine reproductive and respiratory syndrome (PRRS) [5,28]. This may be explained by the different DE miRNAs of the two breeds in response to poly I:C stimulation in the current study, which provided promising genetic evidence for breeders to select individuals with high disease resistance. Similar to miRNA differential expression, in our mRNA expression analysis performed before, out of the 290 and 85 DE genes detected in Dapulian and Landrace, only a small portion of the DE genes (*g* = 33) were shared [20].

MicroRNAs imperfectly bind the 3′-untranslated region (3′-UTR) of their target mRNAs and may cause translation inhibition and/or mRNA degradation [6,29,30]. MicroRNAs can have multiple targets, and a single protein-coding gene can be targeted by multiple miRNAs too. In recent years, several computational methods, based on sequence complementarity and thermodynamic stability of the miRNA and the mRNAs, have been developed, such as TargetScan, miRanda, and Pictar. However, it is known that the results of target prediction algorithms are inconsistent and their expected false positive rates are large [31]. This is mainly caused by two aspects: our understanding of the molecular basis of miRNA-target pairing is relatively limited so far, and post-transcriptional regulation miRNAs are context dependent due to cooperative interactions occurring among different miRNAs. Given the fact of miRNAs acting widely through target degradation, inverse correlations between miRNA expression profiles and those of their target genes could be obtained as expected. The integration of target predictions using algorithms and mRNA gene expression profiles in their common physiological context has been recently proposed to improve the detection of functional miRNA-target relationships [32,33].

In addition to miRNA expression profiling, we also performed mRNA expression profiling of the same samples selected for miRNA profiling [20]. Simultaneous miRNA/mRNA transcriptomes were used here to increase the precision of bioinformatics predictions for miRNA targets. Basing on the filtered target genes, the function analyses indicated that many pathways and GO terms were involved in immune response. Especially, the function analyses of DE target genes, i.e., the differentially expressed target genes in the mRNA transcriptome analysis, were enriched in four immune-related terms, including cellular response to cytokine stimulus (GO:0071345), response to cytokine (GO:0034097), defense response (GO:0006952), and inflammatory response (GO:0006954). All these functional analyses revealed that, by regulating the target genes, DE miRNA played an important role in the regulation of PBMC to poly I:C stimulation.

In order to identify the breed difference in gene expression, we also investigated the breed difference in miRNA expression, including differences in their control groups and poly I:C stimulation groups separately. Consequently, we identified many DE miRNAs in both control groups and poly I:C stimulation groups. Similar to the response to poly I:C stimulation, some of the target genes of these DE miRNAs were enriched in immune-related pathways and GO terms. PBMC is the main immune cell of the whole blood, and it is reasonable that the DE target genes of the DE miRNAs between the breed are mainly enriched in immune-related functions. However, besides the immune-related functions, we found some of the enriched pathways and GO term were around the regulation of basic cellular process, signaling, and metabolic activity, such as general transcription regulation (P00023), Integrin signaling pathway (P00034), G-protein coupled receptor signaling pathway (GO:0007186), and regulation of metabolic process (GO:0019222), which suggested that DE miRNAs between the breeds may be involved in regulation of the different characteristics of the two breeds.

## 4. Material and Methods

### 4.1. PBMC Isolation and Poly I:C Stimulation

In the study, six five-week old pigs were employed as experimental individuals, i.e., three Landrace (two male and one female) and three Dapulian individuals (one male and two female). All piglets did not receive any vaccinations except the classical swine fever (CSF) vaccine on the 21st day after birth. The whole procedure for collection of blood was performed in strict accordance with guideline (IACC20060101, 1 January 2006) of the Institutional Animal Care and Use Committee of Institute of Animal Science and Veterinary Medicine, Shandong Academy of Agricultural Sciences.

About 20 mL peripheral blood was collected per piglet via venipuncture into a vacutainer tube using EDTAK_2_ as anticoagulant. Using Ficoll-Hypaque PLUS (GE healthcare, Sunnyvale, CA, USA), PBMC was isolated by density gradient centrifugation following the manufacturer′s instructions. Then, PBMC, isolated from each piglet, were suspended into 70 mL RPMI-1640 medium (Hyclone, Logan, UT, USA) supplemented with 10% fetal calf serum, 100 mg/mL penicillin, and 100 IU/mL streptomycin. The cell concentration and vitality were finally determined at ~2 × 10^6^/mL and ~95%, respectively. The cell suspension was separated into two units. To one was added poly I:C (Sigma-Aldrich, Munich, Germany) at a concentration of 20 µg/mL to create a stimulated group, and to the other was added the same volume of medium as a control group. Both groups of PBMC were cultured for 24 h at 37 °C with 5% CO_2_. In our previous work, the stimulation with a concentration of 20 µg/mL and the time point of 24 h had been determined as the optimal conditions for achieving the largest immune response [34]. Accordingly, four different experimental groups were generated for the follow-up experiments including DT containing three samples of Dapulian stimulated by poly I:C 24 h, DC containing three samples of Dapulian cultured 24 h, LT containing three samples of Landrace stimulated by poly I:C 24 h, and LC containing three samples of Landrace cultured for 24 h.

### 4.2. Sequencing and Data Processing

For each sample, total RNA was extracted using RNAiso Plus (TaKaRa, Dalian, China) and its quality and amount were measured using a NanoDrop 2000 spectrophotometer (Thermo Scientific, Waltham, MA, USA) and an Agilent 2100 Bioanalyzer (Agilent Technologies, Santa Clara, CA, USA). The RNA integrity number score ≥8 and rRNA 28S/18S ≥1.6 were required in the study. For each sample, ~1 µg of total RNA was used to prepare a small RNA library according to the protocol of TruSeq Small RNA Sample Prep Kits (Illumina, San Diego, CA, USA). Then, for each of the 12 samples, single-end sequencing with a read length of 36 bp was conducted on an Illumina HiSeq2500 following the vendor′s recommended protocol. Sequencing data have been deposited in the National Center for Biotechnology Information Sequence Read Archive with accession no. PRJNA308253 (Available online: http://www.ncbi.nlm.nih.gov/sra/?term= PRJNA308253).

Raw miRNA-seq data was firstly subjected to the Illumina pipeline filter (Solexa 0.3, San Diego, CA, USA), and then the dataset was further processed using ACGT101-miR v4.2 (LC Sciences, Houston, TX, USA), a proprietary pipeline script, to remove adapter dimers, low complexity, repeats, and common RNA families (mRNA, rRNA, tRNA, snRNA, snoRNA). Subsequently, a BLAST search was carried out to align unique sequences ranging from 18 to 26 nucleotides to the porcine precursors involved in miRBase 21.0 (http://www.mirbase.org/) with the criteria of one mismatch inside of the sequence and length variation at both the 3′ and 5′ ends allowed. The unique sequences aligned to porcine mature miRNAs in hairpin arms were identified as known miRNAs, and those aligned to the other arm of the porcine precursor hairpin were considered novel 5 or 3p- derived miRNA candidates. Then, the remaining sequences were mapped to the precursors of all mammal species (with the exclusion of pig) in miRBase 21.0, and the mapped pre-miRNAs were re-aligned to the porcine genome to determine their genomic locations. Finally, the unmapped sequences were BLASTed against the porcine genome, and the hairpin RNA structures were predicted from the flank 80 nt sequences using RNAfold (http://rna.tbi.univie.ac.at/cgi-bin/RNAfold.cgi) with the default folding criteria.

### 4.3. Differentially Expressed miRNA Identification and Their Function Analysis

To detect the differentially expressed miRNAs, each identified miRNA read was normalized using global normalization. If the normalized expression of a certain miRNA was lower than 3 in all 12 individuals, it was excluded from the further differential expression analyses. Multidimensional scaling (MDS) analyses were conducted to visualize the relationships of samples based on all normalized miRNAs by using the function plotMDS from the Bioconductor package “limma” (version 3.20.9) [35]. Differentially expressed miRNAs were determined using *t* testing, and the significance threshold was set to be 0.05 in each test.

Two computational target prediction algorithms, namely miRanda (http://www.microrna.org/microrna/home.do) and TargetScan (http://targetscan.org/), were used to predict the genes targeted by DE miRNAs. Alignment score = 145 and energy = −10 kcal/mol were used for miRanda, while a context score <−0.2 was used for TargetScan. Only high-confidence miRNA targets predicted by both algorithms were used for further analysis.

Pathway and Gene Ontology (GO) enrichment analyses were performed by PANTHER Classification System version 10.0 (www.pantherdb.org) to determine the effects of the predicted target genes, and Bonferroni correction was used to adjust multiple testing to reduce the false positive rate. Additionally, Cytoscape (v3.0.1) [36] was used to create the potential interaction networks of miRNA and their target genes.

### 4.4. Quantitative Real-Time PCR Confirmation

To technically validate the data generated by sequencing, nine miRNAs were selected for further confirmation by quantitative real-time PCR (qPCR) using the miScript PCR System (Qiagen). The miRNA-specific primers for the nine selected miRNA and two endogenous controls were designed using Primer Premier 5.0 (Applied Biosystems, Palo Alto, CA, USA). Briefly, 1 µg of total RNA was reverse-transcribed into cDNA using the miScript II RT Kit of the miScript PCR System following the manufacturer’s directions. The PCR reaction was prepared with the miScript SYBR Green PCR kit of the miScript PCR System and miRNA-specific primers. qPCR assays were performed under the thermal cycling conditions, 95 °C for 5 min, 40 cycles of 94 °C for 15 s, 55 °C for 30 s. All qRT-PCR were carried out using SYBR Green I Master (Roche, Mannheim, Germany) on a Roche LightCycler^®^ 480 instrument following the manufacturer’s guidelines. All samples were run in duplicate for each of the 11 miRNA genes and the reference genes.

The “cor.test” function of R was used to conduct the correlation analysis of the fold change between poly I:C treat and control measured by qPCR and RNA-seq data.

## 5. Conclusions

Overall, a total of 905 miRNAs were identified, of which 503 miRNAs were not annotated in the latest miRBase 21.0. In the expression analysis, we detected 20 and 23 significant DE miRNAs in response to the poly I:C stimulation in Dapulian and Landrace, respectively. Functional analysis revealed that these DE miRNA may play an important regulatory role in the host′s poly I:C stimulation by regulating their target genes. On the other hand, by comparing the two breeds we observed 43 and 61 DE miRNAs in the control groups and poly I:C stimulation groups, respectively, that may be involved in regulating the different characteristics of the two breeds.

## Figures and Tables

**Table 1:** Significant PANTHER pathways for target genes for the differentially expressed (DE) miRNAs between the poly I:C stimulation group and control group of Dapulian (DT_DC) and Landrace (LT_LC).

PANTHER Pathways	Annotated	Dapulian (DT_DC)				Landrace (LT_LC)			
		Significant	Expected	Fold Enrichment	*p*-Value	Significant	Expected	Fold Enrichment	*p*-Value
Angiogenesis (P00005)	147	27	13.37	2.02	6.50 × 10^−^^4^	27	14.56	1.85	2.16 × 10^−^^3^
Cholecystokinin receptor(CCKR) signaling map (P06959)	155	30	14.1	2.13	1.43 × 10^−^^4^	23	15.35	1.5	4.00 × 10^−^^2^
Epidermal growth factor (EGF) receptor signaling pathway (P00018)	134	24	12.19	1.97	1.73 × 10^−^^3^	23	13.27	1.73	9.35 × 10^−^^3^
Fibroblast growth factor (FGF) signaling pathway (P00021)	124	21	11.28	1.86	5.97 × 10^−^^3^	19	12.28	1.55	4.47 × 10^−^^2^
Parkinson disease (P00049)	104	20	9.46	2.11	1.82 × 10^−^^3^	23	10.3	2.23	4.26 × 10^−^^4^
PI3 kinase pathway (P00048)	45	10	4.09	2.44	9.36 × 10^−^^3^	11	4.46	2.47	6.18 × 10^−^^3^
Ras Pathway (P04393)	80	22	7.28	3.02	7.76 × 10^−^^6^	14	7.92	1.77	3.17 × 10^−^^2^
T cell activation (P00053)	83	16	7.55	2.12	4.81 × 10^−^^3^	15	8.22	1.82	2.11 × 10^−^^2^
Toll receptor signaling pathway (P00054)	59	10	5.37	1.86	4.69 × 10^−^^2^	11	5.84	1.88	3.63 × 10^−^^2^
Ubiquitin proteasome pathway (P00060)	65	11	5.91	1.86	3.89 × 10^−^^2^	13	6.44	2.02	1.48 × 10^−^^2^
Gonadotropin releasing hormone receptor pathway (P06664)	223	41	20.28	2.02	3.09 × 10^−^^5^	–	–	–	–
Apoptosis signaling pathway (P00006)	129	24	11.73	2.05	1.06 × 10^−^^3^	–	–	–	–
Fas signaling pathway (P00020)	41	11	3.73	2.95	1.65 × 10^−^^3^	–	–	–	–
Vascaular endothelial growth factor (VEGF) signaling pathway (P00056)	61	13	5.55	2.34	4.71 × 10^−^^3^	–	–	–	–
Integrin signaling pathway (P00034)	153	24	13.92	1.72	8.50 × 10^−^^3^	–	–	–	–
p53 pathway feedback loops 2 (P04398)	53	11	4.82	2.28	1.06 × 10^−^^2^	–	–	–	–
De novo purine biosynthesis (P02738)	46	10	4.18	2.39	1.08 × 10^−^^2^	–	–	–	–
Oxidative stress response (P00046)	29	7	2.64	2.65	1.83 × 10^−^^2^	–	–	–	–
Transforming growth factor beta(TGF-β) signaling pathway (P00052)	90	15	8.19	1.83	2.04 × 10^−^^2^	–	–	–	–
Hypoxia response via HIF activation (P00030)	30	7	2.73	2.57	2.15 × 10^−^^2^	–	–	–	–
Platelet derived growth factor (PDGF) signaling pathway (P00047)	141	21	12.82	1.64	2.17 × 10^−^^2^	–	–	–	–
Interleukin signaling pathway (P00036)	101	16	9.19	1.74	2.56 × 10^−^^2^	–	–	–	–
Inflammation mediated by chemokine and cytokine signaling pathway (P00031)	228	30	20.74	1.45	3.19 × 10^−^^2^	–	–	–	–
Heme biosynthesis (P02746)	14	4	1.27	3.14	4.04 × 10^−^^2^	–	–	–	–
Insulin/IGF pathway-mitogen activated protein kinase kinase/MAP kinase cascade (P00032)	35	7	3.18	2.2	4.35 × 10^−^^2^	–	–	–	–
p53 pathway (P00059)	92	14	8.37	1.67	4.60 × 10^−^^2^	–	–	–	–
Coenzyme A biosynthesis (P02736)	11	–	–	–	–	5	1.09	4.59	5.21 × 10^−^^3^
Salvage pyrimidine ribonucleotides (P02775)	13	–	–	–	–	5	1.29	3.88	1.02 × 10^−^^2^
B cell activation (P00010)	68	–	–	–	–	13	6.74	1.93	2.05 × 10^−^^2^
General transcription regulation (P00023)	34	–	–	–	–	8	3.37	2.38	2.19 × 10^−^^2^
Lysine biosynthesis (P02751)	6	–	–	–	–	3	0.59	>5	2.25 × 10^−^^2^
Insulin/insulin-like growth factor (IGF) pathway-protein kinase B signaling cascade (P00033)	49	–	–	–	–	10	4.85	2.06	2.67 × 10^−^^2^
Angiotensin II-stimulated signaling through G proteins and β-arrestin (P05911)	30	–	–	–	–	7	2.97	2.36	3.20 × 10^−^^2^
Notch signaling pathway (P00045)	40	–	–	–	–	8	3.96	2.02	4.88 × 10^−^^2^

## Supplementary Materials

Supplementary materials can be found at www.mdpi.com/1422-0067/17/10/1601/s1.
